# Chidamide Enhances the Cytotoxicity of Cytarabine and Sorafenib in Acute Myeloid Leukemia Cells by Modulating H3K9me3 and Autophagy Levels

**DOI:** 10.3389/fonc.2019.01276

**Published:** 2019-12-03

**Authors:** He Huang, Yang Wenbing, Aishu Dong, Zhewei He, Rongxing Yao, Wenjian Guo

**Affiliations:** ^1^Department of Hematology, The Second Affiliated Hospital & Yuying Children's Hospital of Wenzhou Medical University, Wenzhou, China; ^2^Department of Emergency, The Second Affiliated Hospital & Yuying Children's Hospital of Wenzhou Medical University, Wenzhou, China; ^3^The Second Clinic College of Wenzhou Medical University, Wenzhou, China

**Keywords:** acute myeloid leukemia, chidamide, H3K9me3, autophagy, SIRT1

## Abstract

Previous studies showed that chidamide enhances the cytotoxicity of drugs in acute myeloid leukemia (AML) cells. Therefore, we examined whether chidamide enhanced the cytotoxicity of drugs in AML cells by affecting H3K9me3 and autophagy levels. AML cells (THP-1 and MV4-11 cells) were treated with chidamide, cytarabine (Ara-c), or sorafenib alone or in combination. Cell proliferation and survival rates were analyzed by MTT, flow cytometry, and Western blotting assays. The results showed that a low dose of chidamide enhanced the cytotoxicity of Ara-c or sorafenib in AML cells, decreasing proliferation and increasing apoptosis. H3K9me3 levels as assessed by Western blotting were upregulated by chidamide treatment. Chromatin immunoprecipitation sequencing, which was used to investigate potential signaling pathways, indicated that the autophagy pathway might play a role in the effects of chidamide. The level of autophagy induced in AML cells upon treatment with Ara-c or sorafenib was inhibited by chidamide, and autophagy markers (LC3, P62) were tested by Western blotting. SIRT1 messenger RNA (mRNA) and protein levels were lower in AML cells treated with Ara-c or sorafenib in combination with chidamide than those in cells treated with these drugs alone. Additionally, the Integrative Genomics Viewer results indicate that the H3K9me3 changes were related to SIRT1-binding sites. Together, these results show that chidamide enhances the cytotoxicity of two chemotherapy drugs in AML cells by increasing the H3K9me3 level and inhibiting autophagy via decreasing the expression of SIRT1. Chidamide may be a potential treatment strategy for AML in the future, especially for refractory AML patients.

## Introduction

Acute myeloid leukemia (AML) is one of the most common malignant clonal diseases of the blood system, with a high mortality rate, high recurrence rate, and high treatment-related mortality rate (1). Strategies for improving AML prognosis are a research priority in the field.

The generally poor prognosis for AML is due to several factors, such as drug resistance, gene mutation, and epigenetic changes. For example, resistance to cytarabine (Ara-c) has been shown to be related to poor prognosis in AML patients (2). Some studies showed that drug resistance of leukemia cells is related to autophagy and demonstrated that autophagy protects leukemia cells from chemotherapy drugs (3). Gene mutation is also related to poor prognosis in AML patients, especially FLT3-ITD gene mutation (4). Recent studies have shown that deacetylation of histones, an epigenetic event, may be one of the worst prognostic factors in malignant diseases, including malignant hematology (5).

Many kinds of histone deacetylase inhibitor (HDACi) have demonstrated therapeutic effects in malignant hematologic diseases (6–9), such as Hodgkin's lymphoma, multiple myeloma, and AML. Moreover, HDACi show tumor cell selectivity and are more cytotoxic to tumor cells than normal cells. Methylation is another common type of histone modification. A previous study demonstrated that a specific histone methylation (histone3 lysine 27, H3K27) mutation is a strong disease accelerator in a RUNX1-RUNX1T1 AML mouse model, suggesting that H3K27me2/3 has an important and selective leukemia-suppressive activity in this genetic context (10). Müller-Tidow et al. (11) demonstrated that widespread changes in other specific histone methylation events (histone H3 lysine 9 trimethylation, H3K9me3) at gene promoters in signature AML histone modification patterns are associated with patient prognosis in AML. Some studies showed that changes in the acetylation of histones can affect the methylation of histones. A recent review (12) highlighted the emerging theme that histone modifications can influence one another, such that one modification recruits or activates chromatin-modifying complexes to generate an additional different histone modification. Lee et al. (13) reported that the histone modification crosstalk included methylation and acetylation. For example, the RNA polymerase II-associated Set2 methyltransferase methylates H3 lysine 36 (K36), creating a mark that targets nucleosomes for H3 and H4 deacetylation by the Rpd3S deacetylase complex after the passage of RNA polymerase.

Chidamide is an HDACi developed in China and is the first oral subtype selective HDACi in the world. Some previous studies including ours have shown that chidamide may be a potential drug for leukemia or myelodysplastic syndromes. We previously found that chidamide synergistically enhanced the apoptosis caused by Ara-c in AML cells (14). Some potential mechanisms include reversing the malignant phenotype and inducing growth inhibition, cell cycle arrest, and extrinsic and intrinsic apoptosis by inhibiting the activity of histone deacetylase enzymes (15, 16). We also found that SIRT1, a histone deacetylase enzyme, modulates autophagy caused by Ara-c in THP-1 and MV4-11 AML cells or sorafenib in MV4-11 AML cells (17).

Based on these studies, we hypothesized that chidamide might affect specific histone methylation (H3K9me3) levels in AML cells that is related to the prognosis of AML and that chidamide might inhibit autophagy by modulating the level of SIRT1.

## Materials and Methods

### Cell Lines

Two AML cell lines, FLT3-ITD^+^ MV4-11 cells and FLT3-ITD^−^ THP-1 cells (resistant to Ara-c), were kindly provided by Professor Ravi Bhatia (City of Hope National Medical Center, Duarte, CA, USA). The MV4-11 cell line was cultured in Iscove's modified Dulbecco's medium (Gibco, Billings, MT, USA) supplemented with 10% fetal bovine serum (Gibco) at 37°C in a humidified incubator containing 5% carbon dioxide. The THP-1 cell line was cultured in Iscove's modified Dulbecco's medium or Roswell Park Memorial Institute-1640 medium (Invitrogen, San Diego, CA, USA) with 10% fetal bovine serum at 37°C in a humidified incubator containing 5% carbon dioxide.

### Chromatin Immunoprecipitation Sequencing

Chromatin immunoprecipitation (ChIP) experiments were performed as described by Kleine-Kohlbrecher et al. (18). Chromatin immunoprecipitation sequencing (ChIP-seq) was based on the Illumina technology sequencing platform; the single/paired-end method was used to complete the ChIP-seq analysis of the two human AML cell lines. Antibody used to bind histones was directed against H3K9me3 (Cell Signaling Technology, Rabbit mAb #13969).

### Drug Treatments

Ara-c and sorafenib were obtained from Selleck Chemicals (Houston, TX, USA). Chidamide was kindly provided by Shenzhen Chipscreen (Shenzhen, China). AML cells were treated with these drugs for 24 h. For THP-1 cells, the dose of Ara-c was 8 μM (14); for MV4-11 cells, the dose of Ara-c was 2 μM (14), and the dose of sorafenib was 60 nM (17). For THP-1 cells, the dose of chidamide was 0.5 μM (14); for MV4-11 cells, the dose of chidamide was 1 μM (14). The dose of these drugs was near the IC50 dose of drugs for AML cells. Sorafenib has no effect on FLT3-ITD^−^ AML cell lines (19) and thus was not used for THP-1 cells. To obtain maximum reduction of cytotoxicity from chidamide, the selected dose was the dose showing the minimum effect on AML cell lines according to our previous study (14).

### Growth Inhibition Assay

Cells were seeded in 96-well-plates at a density of 1 × 10^5^ cells/well. After treatment with different drugs for 24 h, 20 μl of MTT solution (5 mg/ml) was added to each well, and the cells were incubated for an additional 4 h at 37°C. The medium was removed, and 200 μl of dimethyl sulfoxide was added to dissolve the MTT crystals. The plates were read at an absorbance of 570 nm. Cell line experiments were repeated three times.

### Apoptosis Assay

Cells were treated with various drugs for 24 h and then harvested. After being washed twice with phosphate-buffered saline, the cells were resuspended in binding buffer. Cells were then co-stained with 5 μl of fluorescein isothiocyanate-conjugated anti-annexin V and 5 μl of propidium iodide using an apoptosis detection kit (BD Pharmingen, San Diego, CA, USA). The stained cells were analyzed on a FACScan flow cytometer (Becton Dickinson, San Diego, CA, USA) and the software Cell Quest.

### Western Blot Analysis

The cells were harvested, washed twice with phosphate-buffered saline, and lysed in radioimmunoprecipitation assay buffer (Cell Signaling Technology, Beverly, MA, USA) on ice for 30 min. The lysates were centrifuged at 12,000 × g for 15 min at 4°C; the resulting supernatants were collected, and the protein concentrations were determined using bicinchoninic acid reagent. Protein samples (10–20 μg) were separated via 10% sodium dodecyl sulfate polyacrylamide gel electrophoresis gels (Life Technology, USA) and transferred to a polyvinylidene fluoride membrane (Millipore, Billerica, MA, USA). The membranes were blocked in Tris-buffered solution containing 5% non-fat milk for 1 h and then incubated with primary antibodies overnight at 4°C. Primary antibodies against H3K9me3, LC3, P62, and poly (adenosine diphosphate ribose) polymerase (PARP) were obtained from Cell Signaling Technology (Danvers, MA, USA). After being washed with Tris-buffered solution with Tween buffer three times, the membranes were incubated with secondary antibodies for 1.5 h. Protein bands were visualized using an ECL kit (Thermo Scientific, USA) and analyzed using Image Lab software (Bio-Rad, CA, USA).

### Real-Time Reverse Transcription-Polymerase Chain Reaction

Total RNA was extracted using TRIzol (Invitrogen, Carlsbad, CA, USA). Approximately 500 ng of the resulting total RNA was used for reverse transcription reactions. Quantitative polymerase chain reaction (PCR) was performed in triplicate using the SYBR-Green PCR Master Mix kit (Takara, Japan) on an IQ5 real-time PCR instrument (Bio-Rad). The primers sequences were as follows: glyceraldehyde 3-phosphate dehydrogenase, 5′-ATGGGGAAGGTGAAGGTCG-3′ (forward) and 5′-GGGTCATTGATGGCAACAATATC-3′ (reverse); and SIRT1, 5′- TAGCCTTGTCAGATAAGGAAGGA-3′ (forward) and 5′- ACAGCTTCACAGTCAACTTTGT−3′ (reverse).

### Statistical Analysis

Results repeated at least three times were expressed as the mean and standard deviation (mean ± SD). The significance of differences in results, including the proliferation rates, apoptosis rates, protein levels, and mRNA levels, was calculated by one-way analysis of variance, and a *p* < 0.05 was considered to indicate a significant difference.

## Results

### Low Dose of Chidamide Enhanced the Cytotoxic Effect of Chemotherapy Drugs in Acute Myeloid Leukemia Cells

**Figure 1:**
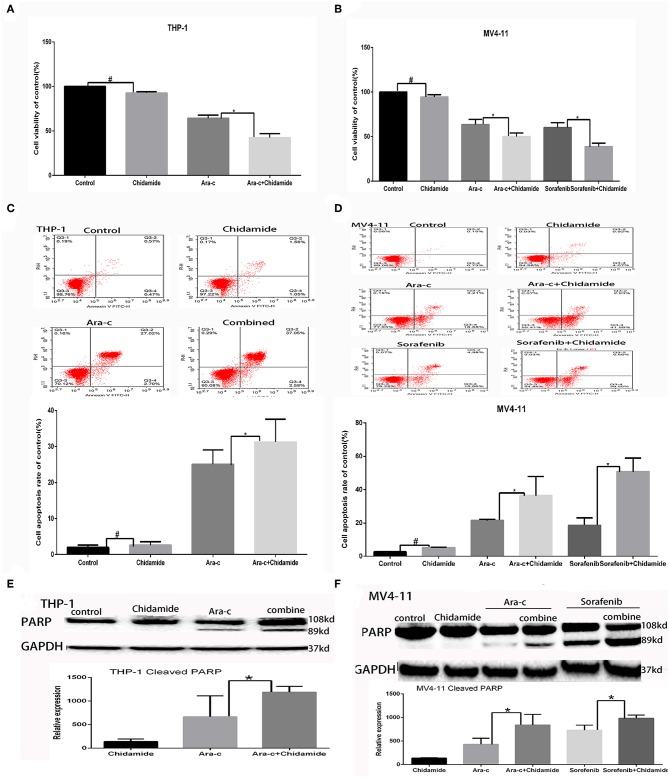
Low-dose chidamide enhanced the cytotoxicity of chemotherapy drugs in acute myeloid leukemia cells. **(A)** MTT assays was performed on THP-1 cells treated with Ara-c alone or in combination with chidamide for 24 h, and the proliferation rate of THP-1 cells in combination with chidamide team was inhibited much more than THP-1 cells treated with Ara-c alone. **(B)** MTT assays was performed on MV4-11 cells treated with Ara-c or sorafenib alone or in combination with chidamide for 24 h, and the proliferation rate of MV4-11 cells in combination with chidamide team was inhibited much more than MV4-11 cells treated with Ara-c or sorafenib alone. **(C)** Flow cytometry assays were performed to evaluate the apoptosis of THP-1 cells treated with Ara-c or in combination with chidamide for 24 h, and the apoptosis rate of THP-1 cells in combination with chidamide team was higher than THP-1 cells treated with Ara-c alone. **(D)** Flow cytometry assays were performed to evaluate the apoptosis of MV4-11 cells treated with Ara-c or sorafenib alone or in combination with chidamide for 24 h, and the apoptosis rate of MV4-11 cells in combination with chidamide team was higher than MV4-11 cells treated with Ara-c or sorafenib alone. **(E)** Western blot analyses were performed on THP-1 cells treated with Ara-c or in combination with chidamide for 24 h treated with Ara-c or in combination with chidamide for 24 h, and the relative expression level of cleaved poly (adenosine diphosphate ribose) polymerase for THP-1 cells in combination with chidamide team was higher than THP-1 treated with Ara-c alone. **(F)** Western blot analyses were performed on MV4-11 cells treated with Ara-c or sorafenib alone or in combination with chidamide for 24 h, and the relative expression level of cleaved poly (adenosine diphosphate ribose) polymerase for MV4-11 cells in combination with chidamide team was higher than MV4-11 cells treated with Ara-c or sorafenib alone. *p < 0.05, ^#^*p* > 0.05.

We performed MTT assays on AML cells (FLT3-ITD^+^ MV4-11 cells and FLT3-ITD^−^ THP-1 cells) treated with various combinations of drugs for 24 h. The proliferation rate for THP-1 cells treated by chidamide only was 91.80 ± 1.97%, and the proliferation rate for MV4-11 cells treated by chidamide only was 94.54 ± 2.49%. The proliferation rate for THP-1 cells treated by Ara-c combined with chidamide was 42.42 ± 4.54%, and the proliferation rates for MV4-11 cells treated by Ara-c or sorafenib combined with chidamide was 50.06 ± 2.06% and 38.80 ± 9.82%, respectively. We found that the proliferation rates were much lower in cells treated with Ara-c or sorafenib in combination with chidamide than those in cells treated with either Ara-c (THP-1 cells was 64.22 ± 3.57%; MV4-11 cells was 63.50±5.80%) or sorafenib alone (MV4-11 cells was 60.19 ± 5.40%). Furthermore, there was no significant change in proliferation rates in cells treated with low-dose chidamide compared with untreated controls (Tables 1, 2 and Figures 1A,B).

**Table 1:** The change in the proliferation of THP-1 cell lines treated by chidamide, Ara-c, and Ara-c combined with chidamide for 24 h.

	**Proliferation rate of AML cell (%)**
THP-1 cells (no drugs treated)	100 ± 0.23
Chidamide	91.80 ± 1.97^#^
Ara-c	64.22 ± 3.57
Combined	42.42 ± 4.54^*^

* *The proliferation rate THP-1 cell lines treated by Ara-c combined with chidamide for 24 h compared with the proliferation rate of THP-1 cell lines treated by Ara-c, P < 0.05*.

# *The proliferation rate of THP-1 cell lines treated by chidamide for 24 h compared with the proliferation rate of THP-1 cell lines without drugs treated, P > 0.05*.

**Table 2:** The change in the proliferation of MV4-11 cell lines treated by chidamide, Ara-c, sorafenib, and Ara-c combined with chidamide and sorafenib combined with chidamide.

	**Proliferation rate of AML cell (%)**
MV4-11 cells (no drugs treated)	100 ± 0.12
Chidamide	94.54 ± 2.49^#^
Ara-c	63.50 ± 5.80
Ara-c combined with chidamide	50.06 ± 2.06^*^
Sorafenib	60.19 ± 5.40
Sorafenib combined with chidamide	38.80 ± 9.82^*^

* *The proliferation rate of MV4-11 cell lines treated in combined with chidamide for 24 h compared with the proliferation rate of MV4-11 cell lines treated by Ara-c or sorafenib alone, P < 0.05*.

# *The proliferation rate of MV4-11 cell lines treated by chidamide for 24 h compared with the proliferation rate of MV4-11 cell lines without drugs treated, P > 0.05*.

The apoptosis rate for THP-1 cells treated by chidamide only was 3.04 ± 0.47%, and the apoptosis rate for MV4-11 cells treated by chidamide only was 5.18 ± 0.28%. The apoptosis rate for THP-1 cells treated by Ara-c combined with chidamide was 34.37 ± 1.30%, and the apoptosis rate for MV4-11 cells treated by Ara-c or sorafenib combined with chidamide was 36.38 ± 2.62% and 50.83 ± 8.08%, respectively. We also found that the apoptosis rate evaluated by flow cytometry was much higher in AML cells treated with Ara-c or sorafenib in combination with chidamide than that in cells treated with either Ara-c (THP-1 cells was 26.78 ± 2.43%; MV4-11 cells was 21.50 ± 0.55%) or Sorafenib alone (MV4-11 cells was 18.56 ± 4.36%). We did not observe any significant change of apoptosis in cells treated with low-dose chidamide compared with the untreated controls (Tables 3, 4 and Figures 1C,D). Western blot showed that cleaved PARP levels were much higher in cells treated with Ara-c or sorafenib in combination with chidamide than those in cells treated with either Ara-c or sorafenib alone. Similar to the Western blot results, there was no significant change of cleaved PARP level in cells treated with low-dose chidamide compared with that in untreated cells (Figures 1E,F).

**Table 3:** The change in the apoptosis of THP-1 cell lines treated by chidamide, Ara-c, and Ara-c combined with chidamide.

	**Apoptosis rate (%)**
THP-1 cells (no drugs treated)	1.04 ± 0.17
Chidamide	3.04 ± 0.47^#^
Ara-c	26.78 ± 2.43
Combined	34.37 ± 1.30^*^

* *The apoptosis rate of THP-1 cell lines treated by Ara-c combined with Chidamide for 24 h compared with the apoptosis rate of THP-1 cell lines treated by Ara-c, P < 0.05*.

# *The apoptosis rate of THP-1 cell lines treated by Chidamide for 24 h compared with the apoptosis rate of THP-1 cell lines without drugs treated, P > 0.05*.

**Table 4:** The change in the apoptosis of MV4-11 cell lines treated by chidamide, Ara-c, sorafenib, and Ara-c combined with chidamide and sorafenib combined with chidamide.

	**Apoptosis rate (%)**
MV4-11 cells (no drugs treated)	1.78 ± 0.98
Chidamide	5.18 ± 0.28^#^
Ara-c	21.50 ± 0.55
Ara-c combined with chidamide	36.38 ± 2.62^*^
Sorafenib	18.56 ± 4.36
Sorafenib combined with chidamide	50.83 ± 8.08^*^

* *The apoptosis rate of MV4-11 cell lines treated in combined with chidamide for 24 h compared with the apoptosis rate of MV4-11 cell lines treated by Ara-c or sorafenib alone, P < 0.05*.

# *The apoptosis rate of MV4-11 cell lines treated by chidamide for 24 h compared with the apoptosis rate of MV4-11 cell lines without drugs treated, P > 0.05*.

### Chidamide Treatment Increased H3K9me3 Levels in Acute Myeloid Leukemia Cells

**Figure 2:**
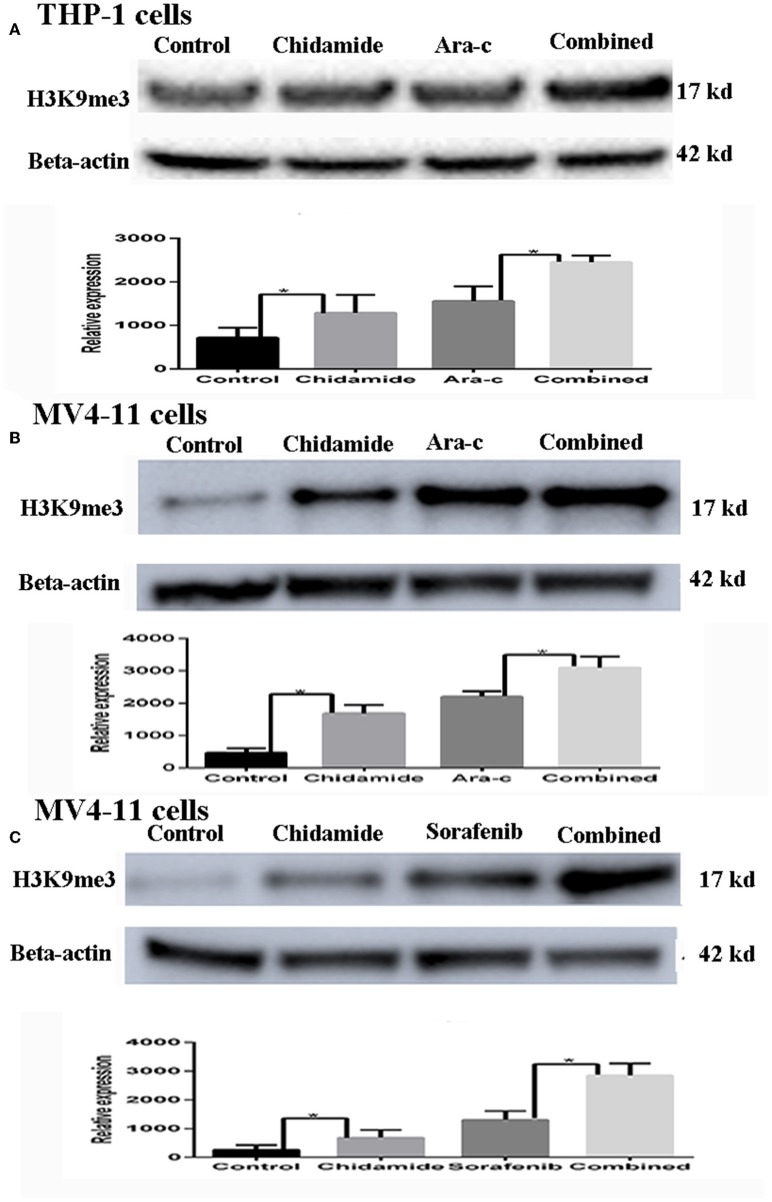
The effect of chidamide on H3K9me3 levels in acute myeloid leukemia cells. **(A)** Western blot analysis of H3K9me3 levels was performed in THP-1 cells treated with Ara-c or in combination with chidamide, and relative expression level of H3K9me3 increased in THP-1 cells treated in combination with chidamide team compared THP-1 cells treated with Ara-c alone. **(B)** Western blot analysis of H3K9me3 was performed on MV4-11 cells treated with Ara-c alone or in combination with chidamide for 24 h, and the relative expression level of H3K9me3 for MV4-11 cells in combination with chidamide team was higher than MV4-11 cells treated with Ara-c alone. **(C)** Western blot analysis of H3K9me3 was performed on MV4-11 cells treated with sorafenib alone or in combination with chidamide for 24 h, and the relative expression level of H3K9me3 for MV4-11 cells in combination with chidamide team was higher than MV4-11 cells treated with sorafenib alone *p < 0.05.

Based on the apoptosis results in AML cells, we selected the same concentration for further experiments. We next evaluated the effect of chidamide on H3K9me3 levels in AML cells by Western blot analysis. H3K9me3 levels were higher in both AML cells treated with chidamide alone compared with those in the untreated controls (Figure 2). Additionally, H3K9me3 levels in cells treated with a combination of drugs were much higher compared with those in the cells treated with only a single drug.

### Chromatin Immunoprecipitation Sequencing Results in Chidamide-Treated Acute Myeloid Leukemia Cells

**Figure 3:**
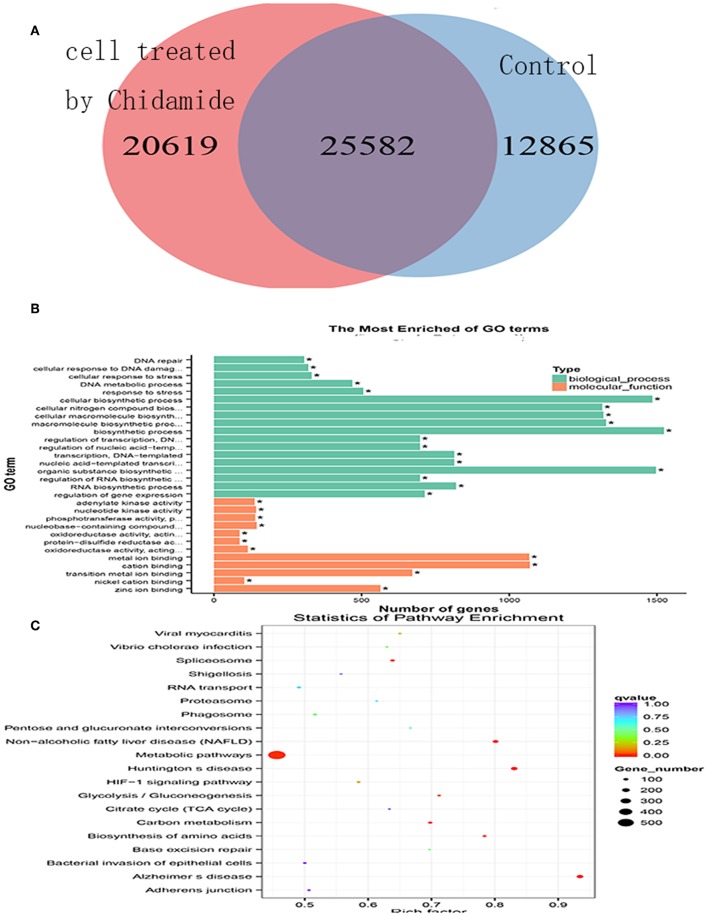
Chromatin immunoprecipitation sequencing in chidamide-treated THP-1 cells. **(A)** A Venn diagram showing the differential expression of H3K9me3 in chidamide-treated THP-1 cells compared with the control (untreated) cells, and there was differential expression in the two teams. **(B)** The results of GO and KEGG enrichment analyses showing the potential changes in signaling pathways for biological processes and molecular functions in chidamide-treated acute myeloid leukemia cells compared with control (untreated) cells. Among the biological processes, we identified several significantly different potential mechanisms, such as DNA repair, cellular response to DNA damage, and stress response. Regarding molecular functions, we also found several significantly different potential mechanisms, such as adenylate kinase activity, and nucleotide kinase activity. **(C)** The results of bubble chart analyses that were performed by GO and KEGG enrichment also showed that the numbers of genes in metabolic pathway were the most.

To investigate the potential mechanism by which chidamide affects AML cells, we next performed ChIP-seq. The results, as displayed in a Venn diagram (Figure 3A), show a differential level of H3K9me3 in AML cells treated with chidamide compared with the untreated group. The peak H3K9me3 levels in the chidamide-treated cells were higher than those in the negative control group, despite a small overlap. GO and KEGG analyses show the potential signaling pathway changes for biological processes and molecular functions in chidamide-treated AML cells compared with the negative control group (Figures 3B,C). Among the biological processes, we identified several significantly different potential mechanisms, such as DNA repair, cellular response to DNA damage, and stress response. Regarding molecular functions, we also found several significantly different potential mechanisms, such as adenylate kinase activity and nucleotide kinase activity.

### Chidamide Treatment Decreased the Level of Autophagy Induced by Chemotherapy Drugs in Acute Myeloid Leukemia Cells

**Figure 4:**
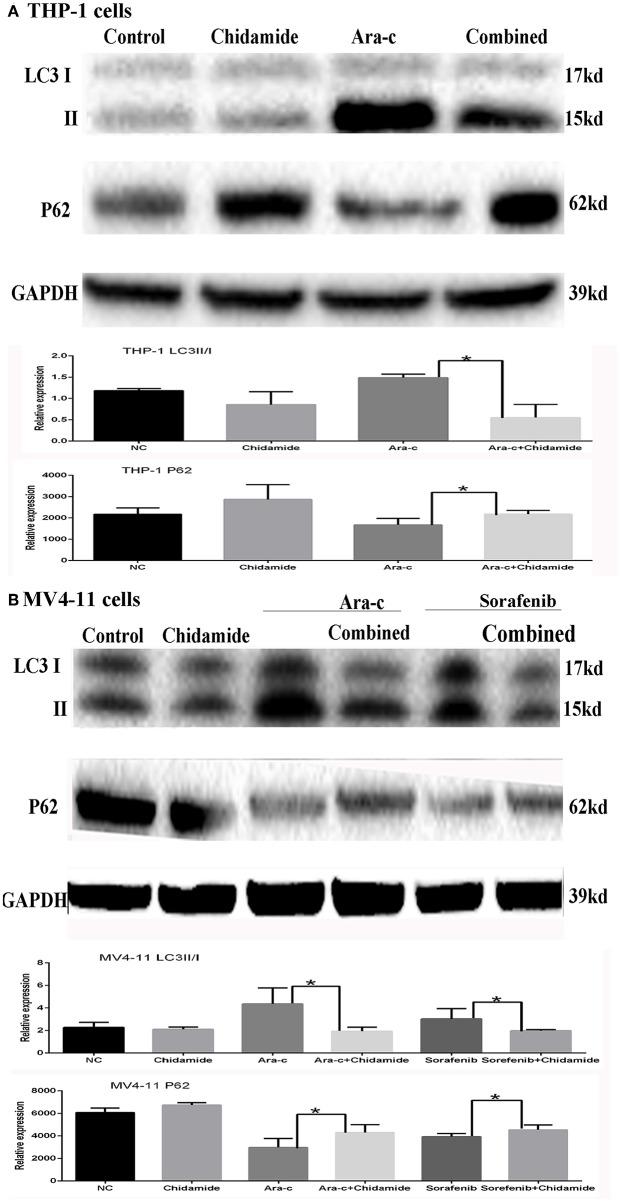
Effect of chidamide on the autophagy level in drug-treated acute myeloid leukemia (AML) cells. Western blot analysis of the p62 protein levels and ratio of LC3II/LC3I in AML cells treated with Ara-c or sorafenib alone or in combination with chidamide, and co-treatment of chidamide with Ara-c or sorafenib in AML cells, the level of p62 protein increased, and the ratio of LC3II/LC3I decreased, indicating that chidamide inhibited the Ara-c- or sorafenib-induced level of autophagy in AML cells. **(A)** The level of p62 protein increased and the ratio of LC3II/LC3I decreased, indicating that chidamide inhibited the Ara-c induced level of autophagy in THP-1 cells. **(B)** The level of p62 protein increased and the ratio of LC3II/LC3I decreased, indicating that chidamide inhibited the Ara-c or sorafenib-induced level of autophagy in MV4-11 cells. *p < 0.05.

Based on the GO and KEGG analysis results from the ChIP-seq as well as our previous study on autophagy caused by Ara-c or sorafenib (17), we next examined the effect of chidamide on the inhibition of autophagy in AML cells. We previously showed that treatment of AML cells with Ara-c or sorafenib induced autophagy, as shown by changes in autophagy markers, such as decreased p62 and increased LC3II/LC3I ratio (17). Here, upon co-treatment of chidamide with Ara-c or sorafenib in AML cells, the level of p62 protein increased, and the ratio of LC3II/LC3I decreased, indicating that chidamide inhibited the Ara-c- or sorafenib-induced level of autophagy in AML cells (Figure 4).

### Chidamide Treatment Decreased SIRT1 Expression in Acute Myeloid Leukemia Cells

**Figure 5:**
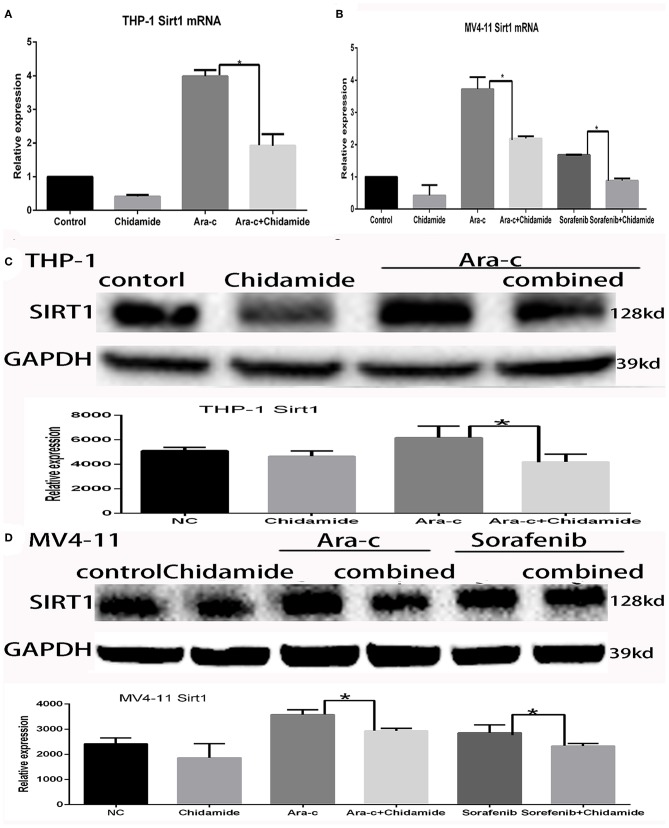
Effect of chidamide on SIRT1 expression in acute myeloid leukemia cells. **(A)** RT-PCR analysis of SIRT1 mRNA levels in THP-1 cells treated with Ara-c alone or in combination with chidamide, and the SIRT1 mRNA levels decreased in THP-1 cells treated in combination with chidamide team compared with THP-1 cells treated with Ara-c alone. **(B)** RT-PCR analysis of SIRT1 mRNA levels in MV4-11 cells treated with Ara-c or sorafenib alone or in combination with chidamide, and the SIRT1 mRNA levels decreased in MV4-11 cells treated in combination with chidamide team compared with MV4-11 cells treated with Ara-c or sorafenib alone. **(C,D)** Western blot analysis of SIRT1 in THP-1 **(C)** or MV4-11 **(D)** cells treated with Ara-c (both cell types) or sorafenib (MV4-11 cells only) alone or in combination with chidamide, and the level of SIRT1 in THP-1 **(C)** or MV4-11 **(D)** cells treated in combination with chidamide team decreased compared with THP-1 **(C)** or MV4-11 **(D)** cells treated with Ara-c (both cell types) or sorafenib (MV4-11 cells only) alone. *p < 0.05.

Previous studies have shown that autophagy is regulated by SIRT1, which induces the translocation of nuclear LC3 to the cytoplasm (20). In our previous research, we identified SIRT1 regulation during autophagy (17); SIRT1 mRNA levels increased in drug-treated cells, while RNA interference-mediated downregulation of SIRT1 resulted in increased p62 and decreased LC3II/LC3I in drug-treated AML cells (17). Here, we found that both SIRT1 mRNA and protein levels were significantly lower in cells treated with chidamide compared with the negative control group (Tables 5, 6 and Figure 5). The relativity of SIRT1 mRNA in THP-1 cells treated by Ara-c combined with chidamide was 1.92 ± 0.34, and the relativity of SIRT1 mRNA in THP-1 cells treated by Ara-c only was 3.98 ± 0.18. The relativity of SIRT1 mRNA in MV4-11 cells treated by Ara-c or sorafenib combined with chidamide was 0.92 ± 0.07 or 2.18 ± 0.15, and the relativity of SIRT1 mRNA in MV4-11 cells treated by Ara-c or sorafenib only was 1.83 ± 0.28 or 3.72 ± 0.34. The SIRT1 mRNA and protein levels were also significantly lower in AML cells treated with Ara-c or sorafenib combined with chidamide compared with cells treated with only Ara-c or sorafenib (Tables 5, 6 and Figure 5). These results suggest that chidamide may inhibit the level of autophagy by decreasing the level of SIRT1 in AML cells.

**Table 5:** The relative value change of SIRT1 mRNA expression in THP-1 cell lines treated by chidamide, Ara-c, and Ara-c combined with chidamide.

	**SIRT1**
THP-1 cells (no drugs treated)	1
Chidamide	0.41 ± 0.04
Ara-c	3.98 ± 0.18
Combined	1.92 ± 0.34^*^

* *The SIRT1 mRNA of THP-1 cell lines treated by Ara-c combined with Chidamide for 24 h compared with the SIR1 mRNA of THP-1 cell lines treated by Ara-c, P < 0.05*.

**Table 6:** The relative value change of SIRT1 mRNA expression in MV4-11 cell lines treated by chidamide, Ara-c, sorafenib, and Ara-c combined with chidamide and sorafenib combined with chidamide.

	**SIRT1**
MV4-11 cells (no drugs treated)	1
Chidamide	0.42 ± 0.34
Ara-c	1.83 ± 0.28
Ara-c combined with chidamide	0.92 ± 0.07^*^
Sorafenib	3.72 ± 0.34
Sorafenib combined with chidamide	2.18 ± 0.15^*^

* *The SIRT1 mRNA of MV4-11 cell lines treated in combined with chidamide for 24 h compared with the SIRT1 mRNA of MV4-11 cell lines treated by Ara-c or sorafenib alone, P < 0.05*.

### The Potential Change in H3K9me3-Binding Sites on SIRT1

**Figure 6:**
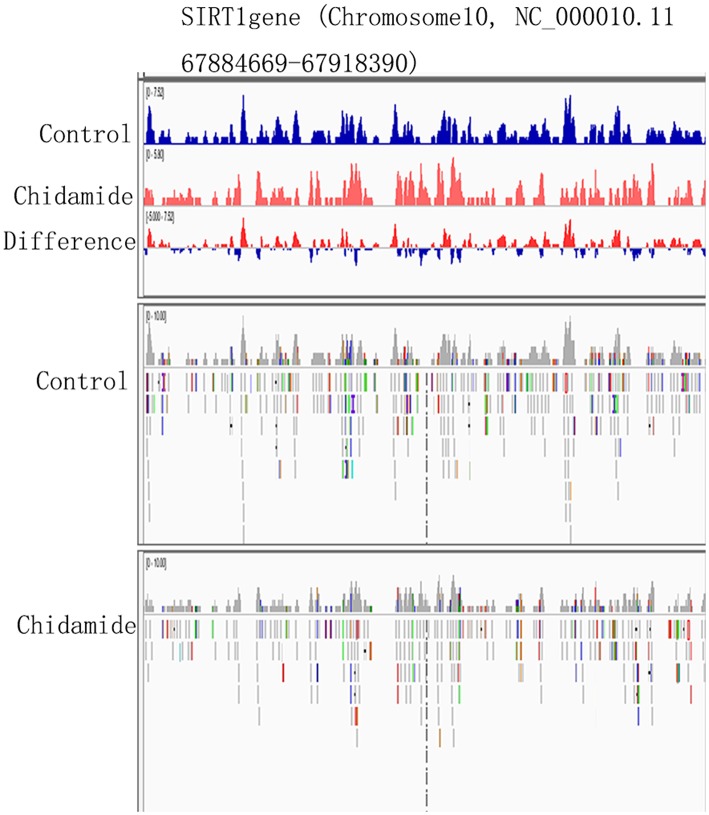
The potential change in H3K9me3-binding sites on SIRT1. The IGV heatmap mode showing the expression level at the same binding site for chidamide-treated acute myeloid leukemia cells and control group cells. The Integrative Genomics Viewer tracking data are also shown.

The ChIP-seq results indicated that some H3K9me3-binding sites are located on chromosome 10. Notably, chromosome 10 also contains the SIRT1 gene (Chromosome10, NC_000010.11 67884669-67918390). Therefore, we hypothesized that the H3K9me3-binding sites on the chromosome 10 might span the SIRT1 gene. The Integrative Genomics Viewer (IGV) results showed that the SIRT1 gene contains some H3K9me3-binding sites (Figure 6); however, the H3K9me3 tracking data for SIRT1 binding sites and expression level were different between chidamide-treated AML cells and untreated cells. The IGV bar-map mode revealed that the expression level for the same binding site was different between chidamide-treated AML cells and control cells. The IGV tracking data also indicated that some changes, like base modification, may exist in these binding sites.

## Discussion

HDACi have been proven to induce cancer cell apoptosis by inhibiting the activity of histone deacetylases in cancer cells, such as lymphoma or breast cancer cells. Chidamide is an HDACi with demonstrated cytotoxicity against T-cell lymphoma. Recently, this compound was also found to be useful for killing cells from other malignant hematological diseases, especially AML. In our previous research, we found that chidamide specifically induced G0/G1 arrest and apoptosis in FLT3-ITD^+^ AML cells in a concentration- and time-dependent manner (14, 15). We also found that chidamide had a cytotoxic effect on FLT3-ITD^+^ and FLT3-ITD^−^ AML cells. Moreover, chidamide showed the same effectiveness in relapsed/refractory AML patients as it did in *de novo* AML patients (14). Another study (16) reported that chidamide inhibited the viability of myelodysplastic syndrome and AML cells by suppressing JAK2/STAT3 signaling or inducing apoptosis via the accumulation of DNA damage and repair defects in AML stem and progenitor cells.

A recent review described mechanisms by which specific histone modifications could influence the levels of other histone modifications. We therefore speculated that chidamide might affect the level of histone methylation during its induction of apoptosis of AML cells via inhibiting the effects of histone deacetylases. A previous study (11) found that H3K9me3 deregulation in AML cells occurred preferentially as a decrease in H3K9me3 levels at core promoter regions. The altered genomic regions showed an overrepresentation of cis-binding sites for ETS and cyclic adenosine monophosphate response elements (CREs) for transcription factors of the CREB/CREM/ATF1 family. The decrease in H3K9me3 levels at CREs was associated with increased CRE-driven promoter activity in AML blasts *in vivo* (11). Based on these reports, we suspected that there might be a change in the H3K9me3 levels of chidamide-treated AML cells. The chidamide cytotoxicity results showed that chidamide alone could not induce apoptosis in AML cells, but it could enhance the cytotoxicity of other chemotherapy drugs, specifically Ara-c and sorafenib, in AML cells. We selected the dose of chidamide that induced the lowest cytotoxicity in AML cells. Chidamide, alone or in combination with other chemotherapy drugs, increased H3K9me3 levels in both THP-1 and MV4-11 cells. These results suggest that chidamide may affect histone methylation. These findings are consistent with those of other studies on the histone modification crosstalk between methylation and acetylation.

To investigate signaling pathway changes that might be related to the observed effects of chidamide on H3K9me3 levels, we conducted ChIP-seq. A Venn diagram of the altered ChIP-seq peaks between the two groups supports the finding that chidamide alters H3K9me3 levels. Furthermore, GO and KEGG analyses revealed some significant changes in signaling pathways for biological processes, such as cellular responses to DNA damage and stress. Notably, the autophagy pathway is related to both the cellular response to DNA damage and the response to stress. Our previous study had showed that the level of autophagy could increase when the drugs (Ara-c or sorafenib) killed AML cells (17), and HDACi have been reported to induce some autophagy-related changes. For example, FK228 (a type I HDACi) induces autophagy and leads to autophagic death in HeLa cells (21). Another study showed that the HDACi SAHA inhibited malignant glioma proliferation in a mouse model by inducing autophagy, which was inhibited by the AKT-mTOR signaling pathway (22). Additionally, the autophagy inhibitor hydroxychloroquine enhanced the cytotoxicity of anticancer drugs by inhibiting SAHA-induced autophagy (23). Other studies also found that HDACi inhibited autophagy by decreasing the acetylation level of autophagy genes and promoting apoptosis (24, 25).

Here, we found that a low dose of chidamide enhanced the cytotoxicity of Ara-c or sorafenib in AML cells, decreasing proliferation and increasing apoptosis. This result is consistent with the idea that a low concentration (below the lethal dose) of HDACi can sensitize tumor cells to additional drug treatments. In our previous research, we found that chemotherapy drugs such as Ara-c or sorafenib also induced autophagy in AML cells, which may be a protective mechanism within the cellular response to DNA damage or stress. Our current data indicate that a low dose of chidamide inhibited the autophagy induced by Ara-c or sorafenib, while a low dose of chidamide by itself showed no significant effects on AML cell proliferation or apoptosis induction. This result suggests a new approach in which chidamide can be used to enhance the cytotoxicity of Ara-c or sorafenib in AML cells and provide further insight into the relationship between histone modification and autophagy.

Based on previous findings on nuclear LC3 and our data on the regulation of autophagy by SIRT1, we speculated that SIRT1 might be involved in the effects of chidamide on AML cells. SIRT1 has the highest homology with yeast Sir2 and targets acetylated lysine residues in both histones and many non-histone proteins for deacetylation to regulate gene expression *in vivo* (25). SIRT1 is involved in cell senescence as well as other physiological activities (25). Over the past decade, SIRT1 has been studied for its important role in tumorigenesis, as it is overexpressed in tumor tissue and tumor cells, such as human AML, and SIRT1 is thought to function as a tumor-promoting factor (26–31). A previous study showed that autophagy in cancer cells is regulated by SIRT1, which induces nuclear LC3 deacetylation and translocation to the cytoplasm (20). LC3 deacetylation in the nucleus is crucial for autophagosome formation, and LC3 is deacetylated in the nucleus by the deacetylase SIRTl during autophagy. The authors further identified that K49 and K51 of LC3 were the main acetylation sites, and deacetylation at these sites was required for LC3 to interact with El-like enzyme Atg7 for subsequent PE conjugation. Together, these data suggest that LC3 deacetylation by SIRT1 in the nucleus is essential for LC3 lipidation and autophagosome formation.

Here, we observed decreased SIRT1 mRNA and protein levels in AML cells upon treatment with chidamide. These results suggest that chidamide inhibits the autophagy induced by Ara-c or sorafenib by downregulating SIRT1 level. Interestingly, ChIP-seq and IGV results revealed H3K9me3-binding sites on chromosome 10, which also contains the SIRT1 gene. We further found that the H3K9me3-binding sites covered the SIRT1 gene segments and that the H3K9me-binding sites were different between chidamide-treated cells and negative-control cells. The relationship between H3K9me3 and the SIRT1 gene needs further investigation, and determining the mechanism underlying this interaction might yield a new method for AML treatment.

In conclusion, we showed that chidamide enhanced the cytotoxicity of two chemotherapy drugs in AML cells, and the level of H3K9me3 was upregulated in these conditions. This change in H3K9me3 levels may induce autophagy as regulated by the inhibition of SIRT1 expression. Our results support further research on chidamide as a supplemental treatment for AML, especially refractory cases, and may provide strategies for reversing drug resistance in AML cells. Our data also highlight a new direct means of histone modification for inducing the death of AML cells.

## Data Availability Statement

Publicly available datasets were analyzed in this study. This data can be found here: ftp://ftp.ensembl.org/pub/release-91/fasta/homo_sapiens/dna/.

## Author Contributions

HH performed the proliferation, apoptosis, and Western blot assays. YW and ZH performed PCR and ChIP. AD conducted the statistical analysis. RY and WG wrote the paper.

### Conflict of Interest

The authors declare that the research was conducted in the absence of any commercial or financial relationships that could be construed as a potential conflict of interest.
